# *Notes from the Field:* Self-Reported Health Symptoms Following Petroleum Contamination of a Drinking Water System — Oahu, Hawaii, November 2021–February 2022

**DOI:** 10.15585/mmwr.mm7121a4

**Published:** 2022-05-27

**Authors:** Alyssa N. Troeschel, Ben Gerhardstein, Alex Poniatowski, Diana Felton, Amanda Smith, Krishna Surasi, Alyson M. Cavanaugh, Shanna Miko, Michele Bolduc, Vidisha Parasram, Charles Edge, Renée Funk, Maureen Orr

**Affiliations:** ^1^Epidemic Intelligence Service, CDC; ^2^Office of Community Health and Hazard Assessment, Agency for Toxic Substances and Disease Registry, San Francisco, California; ^3^Office of Emergency Management, CDC/ATSDR; ^4^Hawaii Department of Health; ^5^Office of Innovation and Analytics, Agency for Toxic Substances and Disease Registry, Atlanta, Georgia.

In late November 2021, the Hawaii Department of Health (HDOH) received reports from Oahu residents of a fuel-like odor coming from their drinking water (*1*), which was later determined to be related to a November 20, 2021, petroleum (jet fuel) leak at the Red Hill Bulk Fuel Storage Facility. The petroleum leak contaminated the Joint Base Pearl Harbor-Hickam water system,* which supplies an estimated 9,694 civilian and military households (*2*), in addition to schools and workplaces. HDOH issued a drinking water advisory on November 30, 2021 (*1*), which was not lifted for all affected zones until March 18, 2022.^†^ Persons in thousands of households were offered temporary housing, and alternative drinking water was provided to users of affected water. HDOH requested epidemiologic assistance (Epi-Aid) from CDC/Agency for Toxic Substances and Disease Registry (ATSDR) to assess the incident’s impact on civilian health in the affected area; this was later expanded to include military-affiliated persons.

The team adapted an interviewer-administered survey from the ATSDR Assessment of Chemical Exposures (ACE) toolkit to collect information about potential exposure to contaminated water, health symptoms experienced, and medical care sought. The survey was modified to be self-administered online, similar to a previous ACE investigation (*3*). Persons present in the affected area after the incident were eligible to complete the survey during January 7–February 10, 2022. Parents and guardians completed the survey on behalf of persons aged <18 years. The survey was promoted through electronic and in-person outreach. Household-level response rates were calculated using ArcGIS Pro and U.S. Navy data (*3*). Descriptive statistics were calculated using R software (version 4.1.1; R Foundation). This activity was reviewed by CDC and was conducted consistent with applicable federal law and CDC policy.^§^

A total of 2,289 eligible participants submitted surveys, with at least one household member participating from 1,389 (14%) of 9,694 estimated affected households. The median participant age was 33 years (range = 1–84 years). Participants were predominantly female (59%), non-Hispanic (81%), military-affiliated (88%), and identified their race as White (74%). Among all participants who were residents in the affected area, 1,115 (52%) reported at least one indication that their water was contaminated (i.e., petroleum smell or taste, or visible oily sheen). Participants indicated that they ingested the potentially contaminated water through oral hygiene (1,821; 80%), drinking (1,650; 72%), and cooking (1,629; 71%). Most participants (2,123; 93%) switched to an alternative water source after learning of the incident.

Most participants reported experiencing one or more new or worsened symptoms after the incident (1,980; 87%), many of whom reported symptoms lasting ≥30 days (1,493; 75%). The largest percentages of reported symptoms were those related to the nervous system (62%), followed by the gastrointestinal system (58%), skin (58%), ear, nose, and throat (47%), mental health (46%), eyes (42%), and respiratory system (31%) (Table). Medical care was sought by 853 (37%) of participants after the incident, including 17 who were hospitalized overnight. Among symptomatic participants, 1,591 of 1,980 symptomatic participants (80%) reported improvement in symptoms after switching to an alternative water source. In an open-text field, 53 (2%) participants expressed concerns about possible long-term health effects.

**TABLE:** Occurrence of new or worsened symptoms, and symptoms persisting for ≥30 days after the contamination of a water system by a petroleum leak on November 20, 2021, self-reported by participants of the Hawaii Assessment of Chemical Exposures survey (N = 2,289) — Oahu, Hawaii, November 2021–February 2022

Self-reported symptom	No. (%) of survey participants	
	Experiencing new or worsened symptoms	Experiencing symptoms for ≥30 days*
**Eyes**	967 (42)	514/967 (53)
Increased tearing	498 (22)	303/498 (61)
Irritation/Pain/Burning of eyes	879 (38)	453/879 (52)
**Ear, nose, and throat**	1,078 (47)	553/1,078 (51)
Runny nose	715 (31)	388/715 (54)
Nose bleeds	191 (8)	86/191 (45)
Burning nose or throat	739 (32)	87/739 (12)
Ringing in ears	405 (18)	263/405 (65)
**Nervous system**	1,428 (62)	959/1,428 (67)
Headache	1,318 (58)	726/1,318 (55)
Dizziness/Lightheadedness	875 (38)	463/875 (53)
Seizures/Convulsions	23 (1)	18/23 (78)
Feeling fatigued	1,016 (44)	696/1,016 (69)
Loss of consciousness/Fainting	52 (2)	29/52 (56)
Confusion	424 (19)	271/424 (64)
Difficulty concentrating	738 (32)	530/738 (72)
Difficulty remembering things	644 (28)	483/644 (75)
**Respiratory/Cardiovascular**	719 (31)	463/719 (64)
Chest tightness or pain/Angina	362 (16)	206/362 (57)
Wheezing in chest	189 (8)	126/189 (67)
Difficulty breathing/Feeling out of breath	416 (18)	271/416 (65)
Coughing	522 (23)	303/522 (58)
Burning lungs	185 (8)	107/185 (58)
**Gastrointestinal**	1,332 (58)	566/1,332 (43)
Nausea	929 (41)	391/929 (42)
Vomiting	370 (16)	100/370 (27)
Diarrhea	1,121 (49)	397/1,121 (35)
**Dermatologic**	1,322 (58)	880/1,322 (67)
Irritation/Pain/Burning of skin	859 (38)	476/859 (55)
Skin rash	925 (40)	506/925 (55)
Skin blisters	169 (7)	101/169 (60)
Dry or itchy skin	1,144 (50)	771/1,144 (67)
**Mental health**	1,049 (46)	865/1,049 (83)
Anxiety	839 (37)	667/839 (80)
Agitation/Irritability	696 (30)	549/696 (79)
Difficulty sleeping	744 (33)	590/744 (79)
Feeling depressed	463 (20)	364/463 (79)
Paranoia	226 (10)	179/226 (79)
Tension/Nervousness	656 (29)	524/656 (80)
**Other^†^**	360 (16)	236/360 (66)

* Among those who reported experiencing symptom.

^†^ Participants could report up to four additional symptoms not listed in the symptoms section of the survey.

This novel incident of jet fuel–contaminated drinking water disrupted the lives of thousands of persons. An online survey paired with robust in-person and electronic promotion facilitated rapid information collection from many affected persons across a wide geographic area, including many who were displaced from their homes. This survey method did not allow for prevalence estimates, nor did it capture the full scope of health impacts. Reported symptoms, such as those related to the respiratory system, gastrointestinal tract, nervous system, and mental health, were consistent with previous studies of exposure to petroleum hydrocarbons^¶^ (*4**,**5*), and accounts of some relief from symptoms after switching to an alternative water source support exposure-related health effects. These results highlight the need for preventing exposure to petroleum products and might aid public health professionals and clinicians in detecting and responding to future similar incidents. Exposure levels, duration, and long-term health effects, however, are uncertain. Additional follow-up of the affected population might improve understanding of the overall health impact of this and other petroleum exposure incidents.

## Footnotes

*
https://www.cpf.navy.mil/News/Article/2870459/opening-statements-at-hawaii-state-legislature-briefing/msclkid/opening-statements-at-hawaii-state-legislature-briefing/

†
https://health.hawaii.gov/news/newsroom/doh-declares-four-navy-drinking-water-distribution-system-zones-safe/

§ 45 C.F.R. part 46, 21 C.F.R. part 56; 42 U.S.C. Sect. 241(d); 5 U.S.C. Sect. 552a; 44 U.S.C. Sect. 3501 et seq.

¶
https://wwwn.cdc.gov/TSP/ToxFAQs/ToxFAQsDetails.aspx?faqid=772&toxid=150
